# Increased retinal thickness in sarcoidosis patients with ocular system involvement visualized with optical coherence tomography: a cross-sectional study

**DOI:** 10.1007/s00296-025-05818-2

**Published:** 2025-02-27

**Authors:** Keld-Erik Byg, Torkell Ellingsen, Jimmi Wied, Michella Peiris, Simon Joel Lowater, Tobias Sejbaek, Jakob Grauslund

**Affiliations:** 1 Department of Rheumatology, Odense University Hospital, Odense, Denmark; 2 Rheumatology Research Unit, Department of Clinical Research, University of Southern Denmark, Odense, Denmark; 3 Department of Ophthalmology, Odense University Hospital, Odense, Denmark; 4 Department of Clinical Research, University of Southern Denmark, Odense, Denmark; 5 Department of Neurology, Esbjerg Hospital, University Hospital of Southern Denmark, Esbjerg, Denmark; 6 Department of Regional Health Research, University of Southern Denmark, Odense, Denmark

**Keywords:** Sarcoidosis, Neurosarcoidosis, Ocular, Retina, Optical coherence tomography

## Abstract

This study investigates the thickness of retinal structures in patients with neurosarcoidosis (NS) and ocular sarcoidosis (OS). We compared the central macular thickness (CMT), retinal thickness (RT), central nerve fiber layer (RNFL) thickness, and ganglion cell layer (GCL) thickness using optical coherence tomography. In a cross-sectional study, we categorized 97 sarcoidosis patients (185 eyes) into four groups: patients without ocular or central nervous system sarcoidosis (Non-Ocular/Non-CNS, *n* = 53), patients with OS (Ocular, *n* = 13), patients with NS (CNS, *n* = 16), and patients with combined OS and NS (Ocular/CNS, *n* = 15). The mean age was 51 (14) years. We found no overall difference between the groups in the CMT (*p* = 0.3), RT (*p* = 0.9), RNFL (*p* = 0.3), and GCL measurements (*p* = 0.9). Only in patients with a disease duration of more than five years, the CMT was significantly thicker in the Ocular group (278 μm, *p* < 0.001), the CNS group (267 μm, *p* = 0.04), and the Ocular/CNS group (268 μm, *p* = 0.04), compared to the Non-Ocular/Non-CNS group (249 μm). The RT was significantly thicker in the Ocular group (296 μm, *p* = 0.008) and the Ocular/CNS group (291 μm, *p* = 0.03) compared to the Non-Ocular/Non-CNS group (283 μm). In the RNFL measurements, the Ocular group (33.7 μm, *p* = 0.002) was thicker than the Non-ocular/Non-CNS group (29.1 μm). We found an increased retinal thickness in patients with ocular sarcoidosis and long disease duration.

## Introduction

Sarcoidosis is a multisystem inflammatory disorder with unknown etiology, causing non-necrotizing granulomas in the affected organs [1]. The incidence in Denmark is 7–15 per 100,000 person-years [2, 3]. The main sites of manifestation are the lungs and intrathoracic hilar lymph nodes. However, all other organs can be involved, which may give rise to central nervous system sarcoidosis, neurosarcoidosis (NS), and ocular sarcoidosis (OS) [4].

NS has a variety of clinical presentations depending on the localization of the granulomatous inflammation and is often part of the initial appearing symptoms [5]. Typical symptoms are cranial neuropathy involving the facial and optic nerves [6], headache, and peripheral motor and sensor signs [7, 5]. Likewise, all structures can be involved in OS. Still, bilateral anterior uveitis is the most common clinical appearance [8, 9].

Optical coherence tomography (OCT) is a well-established, non-invasive method using echo-delay of back-reflected infrared light to produce high-resolution cross-sectional tissue images of retinal layers [10]. It is used in diagnosing and monitoring response or progression to treatment in many retinal diseases [11].

In sarcoidosis, OCT has been used to examine for macular edema, granulomas, macular vessel density, and retinal and choroidal vascularity [12–16]. Furthermore, choroidal thickness has been reported to decrease subfoveal but not peripapillary in sarcoidosis patients in the quiescent period compared to healthy controls [12, 17]. However, few OCT studies on the thickness of the retinal layer have previously been conducted on sarcoidosis patients [12] and NS patients [18]. No studies have compared retinal thickness between OS and NS patients, and cluster analysis revealed that OS and NS are often seen together [19]. Therefore, this study aims to investigate the potential of quantitative retinal thickness abnormalities by OCT examination to detect subclinical disease activity in a cohort of sarcoidosis patients and to explore a potential link between OS and NS patients.

Our objectives were to quantify and compare (a) central macular thickness (CMT), (b) retinal thickness (RT), (c) retinal nerve fiber layer (RNFL) thickness, and (d) ganglion cell layer (GCL) thickness between four different groups of patients with sarcoidosis; patients without ocular or CNS affection (Non-Ocular/Non-CNS), patients with OS (Ocular), patients with NS (CNS), and patients with combined OS and NS (OS/CNS).

## Methods

### Patients

This observational cross-sectional study was conducted in patients with sarcoidosis. Patients with the International Classification of Disease (ICD10) codes for sarcoidosis (i.e., D86*) were identified in medical journal files from the Department of Rheumatology, Department of Respiratory Medicine, or Department of Opthalmology at Odense University Hospital (OUH), Denmark, between 2008 and 2019. First, we focused on patients with OS or NS. Second, as a sarcoidosis control group, we used sarcoidosis patients without OS and NS. We were aiming at approximately 100 patients in the cohort.

The inclusion criteria comprised adult patients with an age above 18 years, a biopsy showing granulomatous inflammation, and symptoms consistent with sarcoidosis by the American Thoracic Society and European Respiratory Society [20]. To focus on exclusive sarcoidosis retinal layer changes, weexcluded patients with medically treated diabetes, non-sarcoidosis-related ocular and CNS diseases, retinal granulomas, and patients with poor image quality on OCT.

OS was defined as known uveitis within the last ten years based on the Revised Criteria of the International Workshop on Ocular Sarcoidosis (IWOS) [21] and the Standardization of Uveitis Nomenclature (SUN) [22] or a history of vision-impairing optic neuropathy. All NS patients fulfilled the probable or definite NS criteria defined by the Neurosarcoidosis Consortium Consensus group (NCCG) [5].

All patients were examined at the Department of Ophthalmology, OUH.

### Outcome variable

The outcome variables were CMT, RT, RNFL, and GCL thickness measured by OCT.

Macular OCT measurements were performed as defined by the Nomenclature of Optical Coherence Tomography (IN*OCT) panel [10]. The CMT was measured in 1st Early Treatment Diabetic Retinopathy Study (ETDRS) grid (the central grid) and defined as the retinal thickness between the internal limiting membrane (ILM) and the border between the outer segment of photoreceptors (12th layer) and the retinal pigment epithelium /Buch’s membrane (14th layer). The RT was the mean of all 9 ETDRS grids between the layers as the CMT. The RNFL thickness was the mean of all 9 ETDRS grids between the ILM and the border between the RNFL (3rd layer) and the GCL (4th layer). GCL thickness was the mean of all 9 ETDRS grids from the border between the 3rd and 4th layer and the border between the inner plexiform layer (5th layer) and the inner nuclear layer (6th layer).

After pupillary dilation, macular OCT was obtained using the DRI OCT Triton, Swept Source OCT (SS-OCT) (Topcon, Japan) and analyzed using IMAGE Net 6 (Topcon, Japan) within 0.5–1 h after pupillary dilation in a room lightened by artificial light. Most measurements were automated but manually corrected for obvious errors if the lines did not follow the right layers.

### Study factors

Patients were divided into four groups depending on their sarcoidosis phenotype: (a) sarcoidosis patients without ocular or CNS affection (Non-Ocular/Non-CNS), (b) patients with OS (Ocular), (c) patients with NS (CNS), and (d) patients with combined OS and NS (Ocular/CNS).

### Other variables

Information on organ involvement, sex, age, medical treatment, date of the first symptoms, and duration of illness were collected through medical journals and clinical examination of the patients. The ophthalmologic examination at the Department of Ophthalmology included best-corrected visual acuity (BCVA), slit-lamp analysis with gonioscopy, tonometry, and fundus examination.

### Procedures

The same clinician reviewed all the records to categorize patients. Likewise, the same two clinicians examined all patients. Participants’ age was the age at inclusion. Duration of sarcoidosis was defined as the time between biopsy verification of sarcoidosis and inclusion.

The BCVA was assessed using the Early Treatment Diabetic Retinopathy Study chart (Precision Vision, Woodstock, IL, USA) at four meters. Tonometry was done as a screening for raised intra-ocular pressure (IOP > 23 mmHg) and not a structured examination, as different devices were used.

Before dilation, a gonioscopy was performed to visualize peripheral anterior synechia. Afterward, potential active uveitis was examined in mydriasis with tropicamide 10 mg/ml and phenylephrine 10% and classified by the SUN criteria [22].

Data was collected and managed using REDCap electronic data capture tools hosted by Open Patient Data Explorative Network, Odense, Denmark.

### Ethics

Thestudywas approved by the Regional Ethics Committee of Southern Denmark (ID: S-20180153) and the Region of Southern Denmark's record of data processing activities (ID: 19/5546).

All participants signed an informed consent form before enrolment in the study.

### Statistical analysis

Descriptive statistics were calculated for all parameters. Continuous data was presented as either mean and standard deviation (SD) or median with interquartile range (IQR). One-way-ANOVA or the Kruskal–Wallis test was used to compare groups depending on whether the data were normally distributed. Categorical data were reported as counts (n) and proportions (%) and compared using Fisher’s exact test.

The continuous variables, CMT, RT, RNFL, and GCL were analyzed using linear regression with cluster robust standard error, as every participant could contribute with two eyes, with sarcoidosis phenotype as categorical variables. The individual groups were compared to the Non-ocular/non-CNS group. Adjustments for age, sex, and immunosuppression did not improve the statistical model and were left out of the final model. Furthermore, we did a sub-analysis for patients who had been ill for less or more than five years.

A p-value below 0.05 was considered statistically significant. No correction for multiple analyses was made.

### *Open* data sharing

The datasets generated during and analyzed during the current study are available from the corresponding author upon reasonable request. Relevant authorities, e.g., the Danish Data Protection Agency, must approve the data requestors in adherence to GDPR regulations.

## Results

Of the 170 patients invited, 106 were included, and 97 patients participated with 185 eyes. Our inclusion rate was 62%. The mean age of the non-responders was 47 years, and 53% were men. We excluded nine participants: withdrew consent (*n* = 2), diabetes (*n* = 1), other ocular or CNS diseases (*n* = 4), poor image quality or eye surgery (*n* = 2). Furthermore, nine patients attended with only one eye: one-eyed (*n* = 1), papillary granuloma (*n* = 1), epiretinal fibrosis, and poor image quality (*n* = 7).

At inclusion, the mean (SD) age was 51 (14) years, 53% were males, and the median (IQR) duration of sarcoidosis was 5 (3, 9) years. The participants were distributed as follows: non-Ocular/non-CNS group (*n* = 53), Ocular group (*n* = 13), CNS group (*n* = 16), and Ocular/CNS group (*n* = 15) (Table 1). The participants had significantly different ETDRS scores (*p* = 0.001), and the CNS group had a higher age (*p* = 0.02). The groups did not differ according to sex (*p* = 0.6) or duration of sarcoidosis (*p* = 0.3).

**Table 1:** Baseline characteristics for four different sarcoidosis phenotypes

Characteristic	Non-Ocular/ Non-CNS^a^ group	Ocular^b^ group	CNS^c^ group	Ocular/CNS^d^ group	*p*-value
N	53	13	16	15	
Age, years	49 (12)	54 (15)	59 (12)	45 (15)	0.02^h^
Sex, % male	51	46	69	60	0.6^i^
Duration of sarcoidosis, years	5 (3, 9)	7 (3, 9)	9 (5, 13)	5 (3, 9)	0.3^j^
<2 years, n (%)	8 (15)	2 (15)	1 (6)	2 (13)	0.9^i^
2–5 years, n (%)	22 (42)	4 (31)	5 (31)	7 (47)	
>5 years, n (%)	23 (43)	7 (54)	10 (63)	6 (40)	
Immunosuppression, n (%)	39 (74)	12 (92)	15 (94)	11 (73)	0.2^i^
Prednisolone, n (%)	18 (34)	8 (62)	9 (56)	8 (53)	0.2^i^
DMARD^e^, n (%)	27 (51)	11 (85)	11 (69)	5 (33)	0.03^i^
TNF^f^ inhibitor, n (%)	2 (4)	0	1 (6)	1 (7)	0.8^i^
ETDRS^g^ score, letters	87 (84, 89)	84 (77, 86)	86 (83, 89)	89 (83, 90)	0.001^j^

Values are in count and percentage, mean and standard division (SD), or median and interquartile range (IQR). ^a^ Non-Ocular/Non-CNS group: patients without ocular sarcoidosis or CNS sarcoidosis; ^b^ Ocular group: patients with ocular sarcoidosis; ^c^ CNS group: patients with neurosarcoidosis; ^d^ Ocular/CNS group: patients with combined ocular sarcoidosis and neurosarcoidosis. ^e^ DMARD: methotrexate, azathioprine, mycophenolate mofetil. ^f^ Tumor Necrosis Factor. ^g^ ETDRS: Early Treatment Diabetic Retinopathy study. ^h^ p-values by one-way ANOVA; ^i^ Fisher’s exact test; ^j^ Kruskal-Wallis Test

Among the patients with ocular involvement, three had active uveitis at the time of the examination; two were in the Ocular group, and one was in the Ocular/CNS group.

Most participants (Table 1) were on immunosuppression (73–94%) without a difference between the four groups (*p* = 0.2). However, there was a significant difference (*p* = 0.03) in participants receiving disease-modifying antirheumatic drugs (DMARD) methotrexate (*n* = 39), azathioprine (*n* = 10), mycophenolate mofetil (*n* = 5), in the non-Ocular/non-CNS group (51%), the Ocular group (85%), the CNS group (69%), and the Ocular/CNS group (33%). Four participants were receiving TNF inhibition. In addition, for patients with sarcoidosis for more than five years, we found no difference in the frequency of patients receiving immunosuppression (*p* = 0.4).

**Fig. 1:**
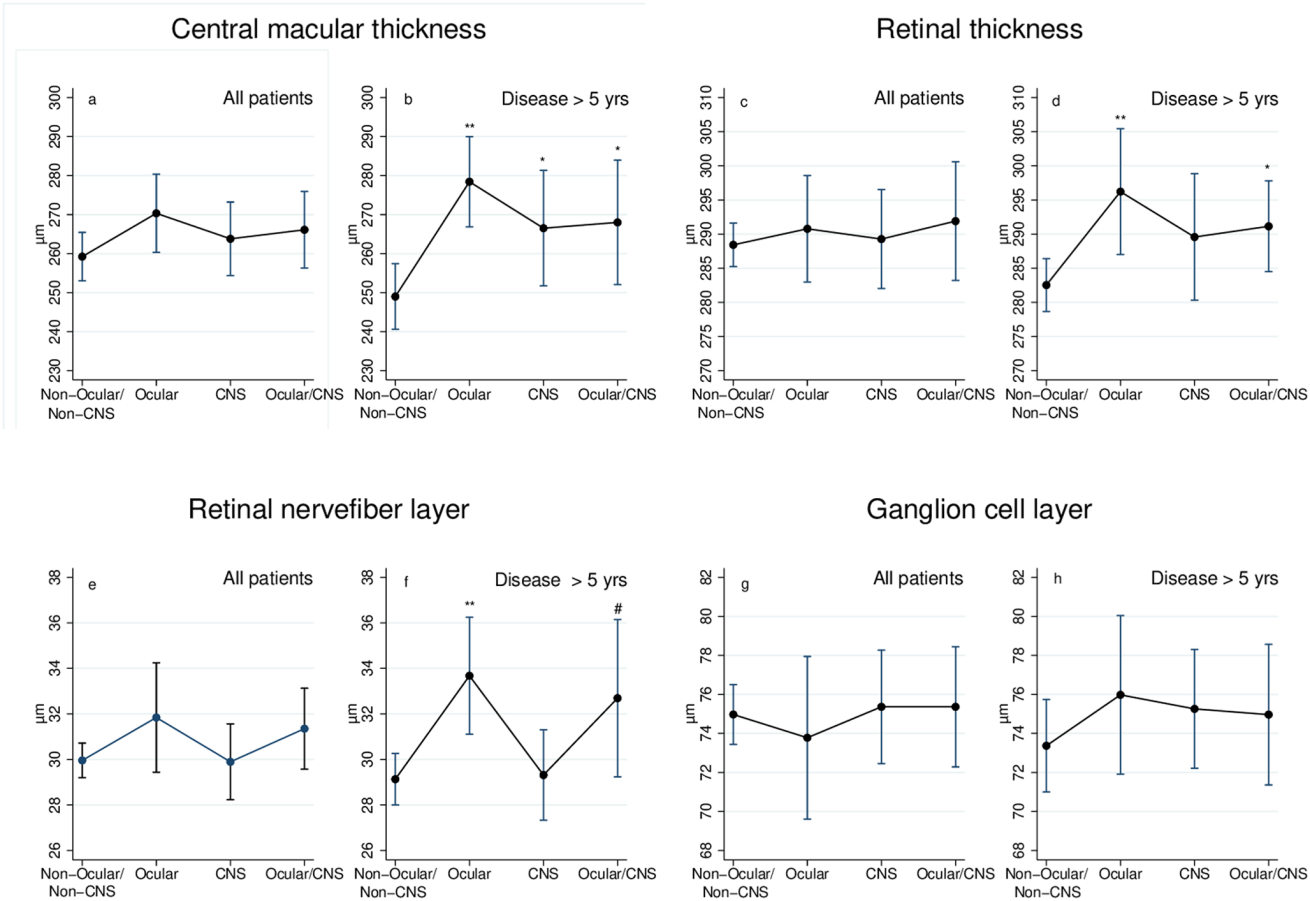
Retinal layer thicknesses in eyes from four different sarcoidosis phenotypes. Non-Ocular/Non-CNS: patients without ocular sarcoidosis or CNS sarcoidosis; Ocular: patients with ocular sarcoidosis; CNS: patients with neurosarcoidosis; Ocular/CNS: patients with combined ocular sarcoidosis and neurosarcoidosis. ** *p* < 0.01; * *p* < 0.05; # *p* = 0.06

Looking at the thickness of the retinal layer (Table 2), we found no difference between the groups in the CMT (*p* = 0.3), RT (*p* = 0.9), RNFL (*p* = 0.3), and GCL measurements (*p* = 0.9) (Fig. 1a, c, e and g). The same was the case when looking at participants with a duration of sarcoidosis of five years or less.

**Table 2:** Predicted retinal layer thickness in eyes from four different phenotypes of sarcoidosis

Characteristic	Non-Ocular/ Non-CNS^a^ group	Ocular^b^ group	CNS^c^ group	Ocular/CNS^d^ group	*p*-value
N (eyes)	105	24	30	26	
CMT	259 (3)	270 (5)	264 (5)	266 (5)	0.3
RT	288 (2)	291 (4)	289 (4)	292 (4)	0.9
RNFL	30.0 (0.4)	31.8 (1.2)	29.9 (0.8)	31.4 (0.9)	0.3
GCL	75.0 (0.8)	73.8 (2.1)	75.4 (1.5)	75.4 (1.6)	0.9
Length of disease ≤ 5 years, N (eyes)	59	10	11	17	
CMT	267 (4)	259 (7)	259 (3)	265 (6)	0.4
RT	293 (2)	283 (6)	289 (6)	292 (7)	0.5
RNFL	30.6 (0.5)	29.3 (1.7)	30.9 (1.4)	30.6 (0.9)	0.9
GCL	76.2 (1.0)	70.7 (4.0)	75.5 (3.1)	75.6 (2.2)	0.6
Length of disease > 5 years, N (eyes)	46	14	19	9	
CMT	249 (4)	278 (6) *P* < 0.001	267 (7) *p* = 0.04	268 (8) *p* = 0.04	0.001
RT	283 (2)	296 (5) *p* = 0.008	290 (5) *p* = 0.2	291 (3) *p* = 0.03	0.02
RNFL	29.1 (0.6)	33.7 (1.3) *P* = 0.002	29.3 (1.0) *p* = 0.9	32.7 (1.7) *P* = 0.06	0.01
GCL	73.4 (1.2)	76.0 (2.0)	75.3 (1.5)	75.0 (1.8)	0.6

Values of the predicted mean and standard error of the mean in µm. ^a^ Non-Ocular/Non-CNS group: patients without ocular sarcoidosis or CNS sarcoidosis; ^b^ Ocular group: patients with ocular sarcoidosis; ^c^ CNS group: patients with neurosarcoidosis; ^d^ Ocular/CNS group: patients with combined ocular sarcoidosis and neurosarcoidosis. CMT: Central macular thickness; RT: retinal thickness; RNFL: retinal nerve fiber layer; GCL: ganglion cell layer

For patients with sarcoidosis for more than five years, the CMT (Fig. 1b) was significantly thicker in the Ocular group (278 μm, *p* < 0.001), the CNS group (267 μm, *p* = 0.04), and the Ocular/CNS group (268 μm, *p* = 0.04), compared to the Non-Ocular/Non-CNS group (249 μm). The RT (Fig. 1d) was significantly thicker in the Ocular group (296 μm, *p* = 0.008) and the Ocular/CNS group (291 μm, *p* = 0.03), but not in the CNS group (290 μm, *p* = 0.2) compared to the Non-Ocular/Non-CNS group (283 μm). In the RNFL (Fig. 1f) measurements, the Ocular group (33.7 μm, *p* = 0.002) was thicker than the Non-Ocular/Non-CNS group (29.1 μm). The difference was insignificant in the Ocular/CNS group (32.7 μm, *p* = 0.06). We found no difference compared to the CNS group (29.3 μm, *p* = 0.9) or between the groups in the GCL measurements (Fig. 1h).

## Discussion

In this observational cross-sectional study, we investigated quantitative OCT to measure retinal abnormalities in patients with sarcoidosis with four different phenotypes: a Non-Ocular/Non-CNS group, an Ocular group, a CNS group, and an Ocular/CNS group.

In patients with an illness lasting more than five years, we found a change in retinal thickness. Compared to the Non-Ocular/Non-CNS group, the CMT was thicker in patients with an OS or NS phenotype (Ocular group, CNS group, Ocular/CNS group). Furthermore, in the patients with OS phenotypes (Ocular group, Ocular/CNS group), we also found an increased thickness in RT and the same tendency in RNFL.

OCT has been used as a potential biomarker for diagnostics and investigation of the severity and progression of neurodegenerative disorders like multiple sclerosis (MS), Parkinson’s disease, and Alzheimer’s disease. The RNFL, macular, and GCL thickness was generally reduced, even in MS patients without a history of optic neuritis [23, 24].In systemic rheumatologic diseases, patients with systemic lupus erythematosus over ten years had reduced macular thickness [25]. However, RNFL thickness in fibromyalgia syndrome and systemic sclerosis patients was not different compared to healthy controls (HC) [26, 27]. In contrast, for patients with Vogt–Koyanagi–Harada Disease, a higher median RNFL was found among subjects who developed chronic and recurrent disease. Yet, the RNFL measurements were conducted after two months post-treatment [28].

Only a few studies have investigated retinal thickness in patients with sarcoidosis. Ugurlu et al. [12] examined patients with sarcoidosis without a uveitis history or active signs of uveitis and found no difference in peripapillary RNFL thickness compared to a healthy control group. In contrast, Eckstein et al. [18] investigated 20 patients with NS. They found that 75% of patients with NS with ocular symptoms and 33% of patients with NS without ocular symptoms demonstrated quantitative OCT abnormalities compared to healthy controls. The changes could be RNFL and macular thinning and swelling, and the OCT abnormalities did not differ according to the NS subtype or cerebrospinal fluid findings. In addition, the average macular thickness was inversely correlated with disease duration. However, while they focused on peri-papillary measurement, their results were not directly comparable to our results.

In contrast, we found increased retinal thickness in patients with NS and OS phenotypes with a long disease duration of five years or more. The CMT was thicker in all NS and OS phenotypes; the RT was thicker in the Ocular and Ocular/CNS groups, and the RNFL was thicker in the Ocular group. The retinal changes in our study may represent primary retinal changes, as there was no significant difference in patients receiving immunosuppression, we had removed all patients with visible retinal granulomas, and optic neuritis causes RNFL thinning after the acute phase [29, 30]. In addition, the increase in the CMT thickness in the CNS group may also represent subclinical ocular inflammation in patients with NS. Quiescent uveitis could be an explanation for our findings. Moore et al. [31] showed RNFL thickness in both active and quiescent uveitis in a mixed group of uveitis patients, including patients with sarcoidosis. In contrast, in a mixed group of uveitis patients without sarcoidosis patients, Lee et al. [32] showed CMT and RNFL thickness only during the acute phase of uveitis.

Based on the same study population, we have previously looked at retinal oxygen metabolism, showing that this was increased in patients with combined OS and NS [33]. In combination, these findings indicate that OS and NS patients might have a subclinical retinal affection, which conventional screening methods cannot recognize.

The strengths of our study include the single-center study design with a large cohort of well-defined, biopsy-verified patients with sarcoidosis stratified based on solid objective criteria. In addition, all patient examinations were done by the same investigators.

The study comprises several limitations. First, the cross-sectional study design made it impossible to determine underlying causality. Second, there was no HC group. An HC group would have validated the result better. Third, even in a highly specialized University Hospital, the number of NS and OS patients is limited in the subgroups, which makes it difficult to generalize the result. Fourth, most patients were taking immunosuppressant medicine, which may suppress the intensity of the disease and, therefore, not count the full effect of the inflammatory process. Finally, although the assessment of the OCT scans did not reveal granulomas, their presence cannot be denied entirely, and hence, they contribute to an increase in macular thickness.

This study demonstrated an increased retinal thickness in patients with ocular sarcoidosis and neurosarcoidosis with long disease duration, examined by OCT. The study indicates the existence of subclinical retinal inflammation that is not detectable with conventional ophthalmological examinations, thus highlighting that OCT may aid as a non-invasive method for visualization of potential CNS and ocular affection. Further studies are required to evaluate OCT as a screening tool for inflammation, disease activity, and prognosis.

## Declarations

### Disclaimer

Preliminary analysis of the data has been published as a congress abstract: Kindt A, Byg K-E, Wied J, Ellingsen T, Rømhild Davidsen J, Grauslund J (2021). Increased retinal thickness in patients with ocular sarcoidosis. Invest Ophthalmol Vis Sci. 2021;62:2508. AI has not been used to write and edit any part of this manuscript, including the text and graphics.
